# Optimal combination of electrodes and conductive gels for brain electrical impedance tomography

**DOI:** 10.1186/s12938-018-0617-y

**Published:** 2018-12-20

**Authors:** Lin Yang, Heng Li, Junjie Ding, Weichen Li, Xiuzhen Dong, Zhihong Wen, Xuetao Shi

**Affiliations:** 1 Department of Aerospace Medicine, Fourth Military Medical University, Xi’an, China; 2 Department of Biomedical Engineering, Fourth Military Medical University, Xi’an, China; 3 School of Preclinical Medicine, Fourth Military Medical University, Xi’an, China

**Keywords:** Brain EIT, Electrode, Conductive gel, Combination

## Abstract

**Background:**

Electrical impedance tomography (EIT) is an emerging imaging technology that has been used to monitor brain injury and detect acute stroke. The time and frequency properties of electrode–skin contact impedance are important for brain EIT because brain EIT measurement is performed over a long period when used to monitor brain injury, and is carried out across a wide range of frequencies when used to detect stroke. To our knowledge, no study has simultaneously investigated the time and frequency properties of both electrode and conductive gel for brain EIT.

**Methods:**

In this study, the contact impedance of 16 combinations consisting of 4 kinds of clinical electrode and five types of commonly used conductive gel was measured on ten volunteers’ scalp for a period of 1 h at frequencies from 100 Hz to 1 MHz using the two-electrode method. And then the performance of each combination was systematically evaluated in terms of the magnitude of contact impedance, and changes in contact impedance with time and frequency.

**Results:**

Results showed that combination of Ag^+^/Ag^+^Cl^−^ powder electrode and low viscosity conductive gel performed best overall (Ten 20^®^ in this study); it had a relatively low magnitude of contact impedance and superior performance regarding contact impedance with time (*p* < 0.05) and frequency (*p* < 0.05).

**Conclusions:**

Experimental results indicates that the combination of Ag^+^/Ag^+^Cl^−^ powder electrode and low viscosity conductive gel may be the best choice for brain EIT.

## Background

Electrical impedance tomography (EIT) is a computed tomography technique that has been used to show the spatial distribution of electrical impedance inside the human body. In EIT, small currents are injected into the human body through surface electrodes attached to the body, and simultaneously the boundary voltages are collected to reconstruct an impedance-based image [1]. Although EIT suffers from a relatively low spatial resolution compared with other existing imaging techniques, such as CT and MRI, it provides several unique advantages in terms of low cost, non-invasion, dynamic image monitoring and functional imaging [2]. Since the impedance properties of biological tissues differs for various physiological and pathological states [3, 4], EIT shows great potential in various clinical scenarios including the detection of breast cancer [5], monitoring pharyngeal and gastric motility [6, 7], and assessing pulmonary ventilation [8] or abdominal bleeding [9].

In addition, there is considerable interest in using EIT to continuously monitor brain injury (such as cerebral edema [10, 11] and intracranial hemorrhage [12, 13]) and rapidly detecting acute stroke [14–16], with the purpose of providing timely and invaluable information for diagnosis and clinical management of patients with brain injury or acute stroke. When real-time monitoring brain injury in the long-term, changes in brain impedance over time caused by brain injury reflect the development of brain injury; this can be used to assess the extent of brain damage [17]. Regarding stroke detection, based on the specific differences in impedance spectra between normal brain tissues and stroke lesions (hemorrhagic and ischemic tissue) [4], changes in brain impedance with frequency can be used to obtain information on stroke lesion, through which the extent and property (hemorrhagic or ischemic) of stroke are evaluated [18].

In brain EIT, electrode–skin contact impedance is a major factor that directly affects the accuracy of measurement because both the current injection and boundary voltage measurement are through the electrode–skin interface. When brain EIT is used in the clinical setting, several conditions must be met to reduce the effects of electrode–skin contact impedance. On the one hand, since the real-time monitoring of brain injury may be implemented over an extended period of time and the electrode–skin contact impedance will change over time due to variations in the conditions (such as temperature and humidity) of the electrode–skin interface, a small change in contact impedance over time (i.e. time property) is required. On the other hand, because multi-frequency currents are employed to obtain the brain impedance spectroscopy when detecting stroke, contact impedance should change as small as possible with frequency (i.e. frequency property). Since the electrode–skin interface consists of the electrode, conductive gel and skin, the behavior of electrode and conductive gel is of crucial importance for contact impedance [19, 20]. Therefore, to improve the quality of measurement when employing brain EIT, time and frequency properties of electrode and conductive gel must be investigated.

Several studies have investigated the performance of the electrodes or conductive gels used in EIT. Xu et al. evaluated the performance of five types of Ag^+^/Ag^+^Cl^−^ electrodes to find the optimal choice for long-term brain EIT monitoring [21]. Puurtinen et al. studied various textile electrodes and concluded that it is feasible to use the textile-based electrodes in physiological monitoring when a conductive gel is applied [22]. Rahal et al. compared of six different types of Ag^+^/Ag^+^Cl^−^ electrodes and a textile electrode for neonate EIT in terms of the impedance characteristics against frequency to identify the best electrode type [23]. Tronstad et al. analyzed the effects of four different types of conductive gel on skin impedance measured at low frequencies (1 Hz–100 kHz) [24]. Additionally, several studies have compared the performance of the electrodes or conductive gels for bio-signal recording. Searle and Kirkup compared the performance of three types of bioelectric recording electrodes (wet, dry and insulating electrode) based on tests involving electrode impedance, static interference and motion artifact for the demand of prolonged recording of bio-signals [25]. Tallgen et al. evaluated the applicability of the different types of commercially available electrodes and conductive gel for slow EEG potential recording, which was done by studying the polarization, initial and long-term stability and low-frequency noise [26]. These studies explored the polarization, noise, long-term stability or frequency property of electrodes or conductive gels for various EIT applications and bio-signal recording; however, to the authors’ knowledge no study has simultaneously investigated the time and frequency properties of combined electrode–conductive gel for brain EIT when brain EIT is used to monitor brain injury in the long-term and/or to rapidly detect stroke.

In this study, four types of clinical electrode and 5 kinds of commonly used conductive gel were selected to obtain 16 combinations of electrode–conductive gel. And then the contact impedance of each combination was measured on the foreheads/scalp of ten healthy volunteer for a period of 1 h at frequencies from 100 Hz to 1 MHz employing the two-electrode strategy; the performance of each combination was evaluated in terms of the magnitude of contact impedance, and changes in contact impedance with time and frequency. Finally, the optimal combination of electrode and conductive gel for brain EIT is discussed.

## Results

None of volunteers showed any discomfort or felt pain throughout the experimental process and measurement conditions were maintained stable for the duration of all the experiments.

### Contact Impedance of electrode–skin

**Fig. 1:**
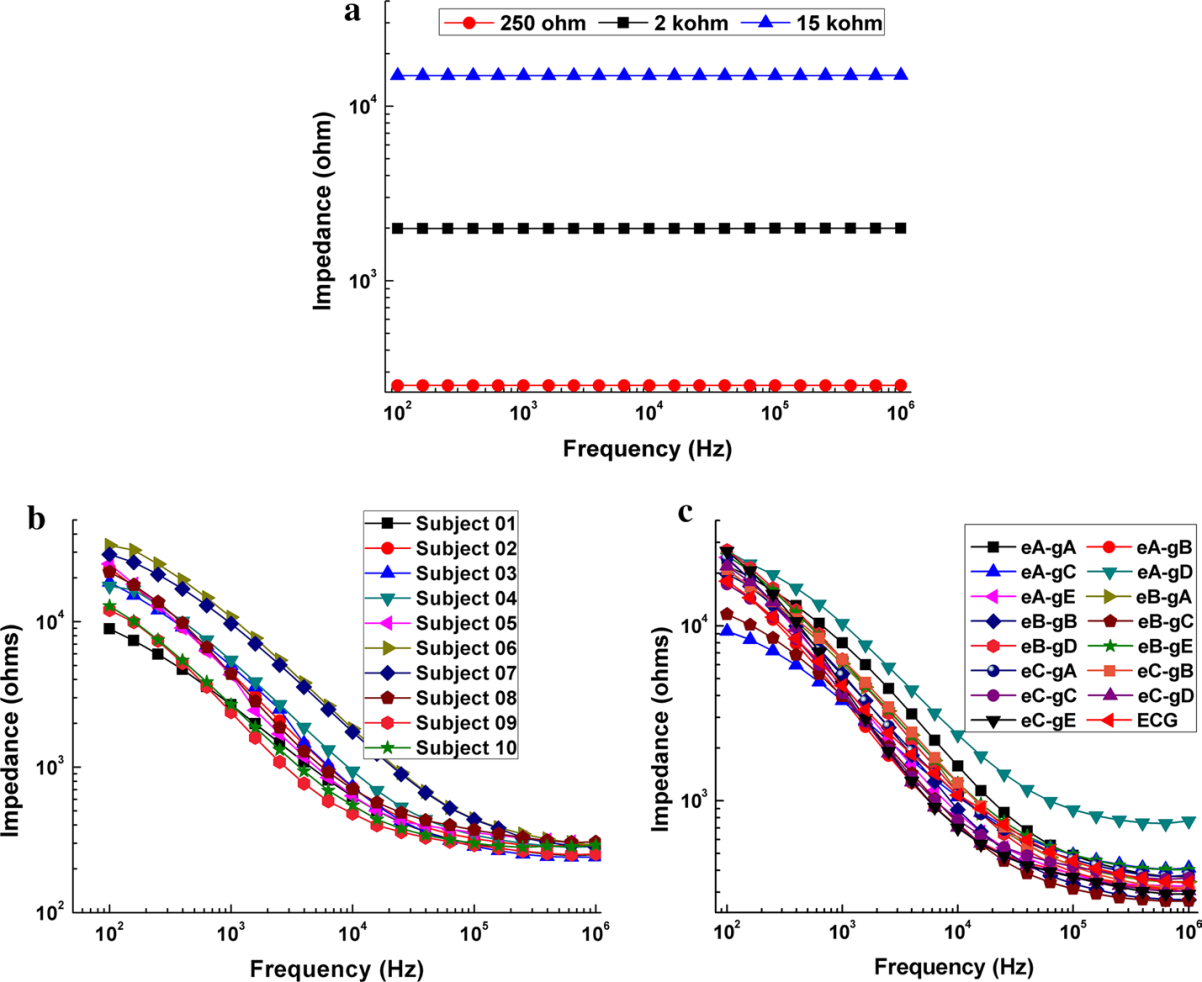
**a** Measurement results of three known resistors (251.12 Ω, 1.99 kΩ and 14.98 kΩ). **b** Measured impedance spectra from 10 volunteers for the combination eA-gA. **c** Mean contact impedances of all combinations of electrode and conductive gel within 100 Hz-1 MHz. eX-gY represents the combination of electrode X and conductive gel Y

Figure 1a shows the measured results of three known resistors—251.12 Ω, 1.99 kΩ and 14.98 kΩ, respectively. The mean of errors for all resistors across the whole frequency range was < 0.2%. This result validated the reliability of our measurements.

Figure 1b exhibits the mean impedance values of all combinations measured immediately after the electrode was applied. The contact impedance quickly decreased with frequency below 100 kHz but slowly with frequencies between 100 kHz and 1 MHz. Within the low frequency range (100 Hz–10 kHz), the mean contact impedance of the eA-gC and eB-gC combinations was clearly small while the mean contact impedance of the eA-gD combination was relatively large. Between 10 kHz and 1 MHz, the mean contact impedance of the eA-gD combination was clearly larger than that of the other combinations, and the mean contact impedance of the eB-gC combination was relatively small.

### Change in contact impedance with time

**Fig. 2:**
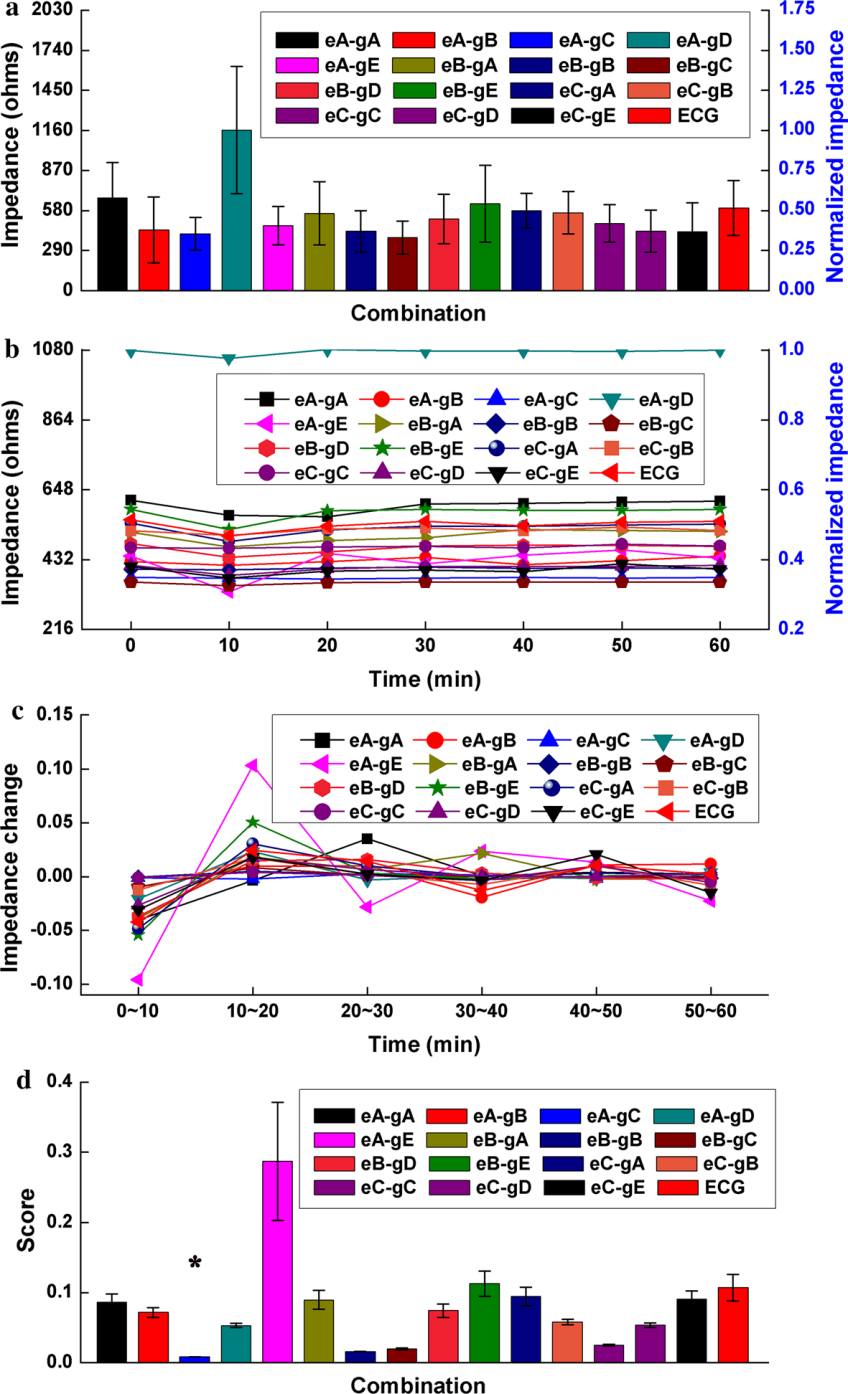
**a** Normalized contact impedance of all electrode–conductive gel combinations measured at 50 kHz. **b** Normalized mean change in contact impedance of all combinations with time measured for 1 h at 50 kHz. **c** Mean change in contact impedance of all combinations during each sub-time frame. **d** Score of all combinations with regard to the time property of contact impedance,*p < 0.05

**Fig. 3:**
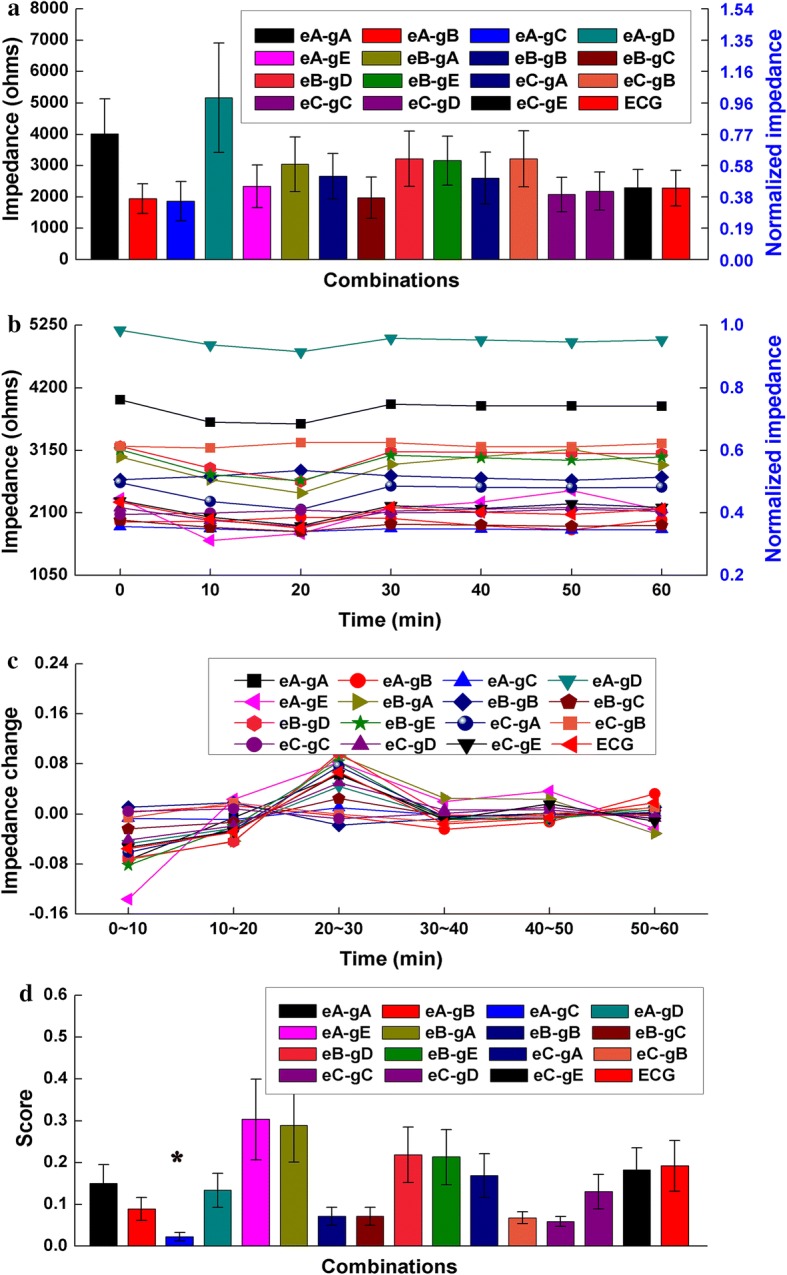
**a** Normalized contact impedance of all electrode–conductive gel combinations measured at 1 kHz. **b** Normalized mean change in contact impedance of all combinations with time measured for 1 h at 1 kHz. **c** Mean change in contact impedance of all combinations during each sub-time frame. **d** Score of all combinations with regard to the time property of contact impedance,*p < 0.05

Figure 2a shows the impedance of all combinations measured at 50 kHz and recorded immediately after applying the electrodes because the current of 50 kHz is often used to monitor intracranial hemorrhage [17, 27–29]. The mean contact impedance of the eB-gC combination appeared to be the smallest, but the discrepancies in contact impedance between eB-gC and the other combinations (except for eA-gD) were not significant (*p* > 0.05). As shown in Fig. 2b, the contact impedance of most combinations obviously varied within about 20 min after the electrodes were applied, while contact impedance remained almost unchanged between 30 and 60 min. Figure 2c shows the change in contact impedance of all combinations during the different sub-time frames. In general, the change in contact impedance of all combinations decreased with time. Figure 2d shows the score of each combination regarding the time property of contact impedance. The eA-gC combination had the smallest score; there were significant differences in score between eA-gC and the other combinations (*p* < 0.05), indicating that the eA-gC combination had the optimal time property for monitoring intracranial hemorrhage. Figure 3 shows the impedance of all combinations measured at 1 kHz because the current at low frequency (< 1 kHz) is usually preferred to monitor intracranial ischemia [12, 30]. The results at 1 kHz were similar to those at 50 kHz and the eA-gC combination had the optimal time property for intracranial ischemia monitoring.

### Change in contact impedance with frequency

Table 1 shows the change in contact impedance of all combinations across the 100 Hz–10 kHz and 10 kHz–1 MHz frequency ranges. It can be seen that the change in contact impedance across the 100 Hz–10 kHz frequency range was much larger compared to the 10 kHz–1 MHz frequency range, indicating that the change in contact impedance across the 100 Hz–10 kHz frequency range was the major contributing factor to the variation in contact impedance across the whole frequency range. At frequencies from 100 Hz to 10 kHz, the change in contact impedance of the eA-gC combination was significantly smaller than that of the other combinations (*p* < 0.05), suggesting that the eA-gC combination had the best frequency performance. Between 10 kHz and 1 MHz, the change in contact impedance of all combinations was similar (*p* > 0.05).

**Table 1:** Change in contact impedance with frequency of all electrode–conductive gel combinations

Frequency range	Impedance change of different combinations (kΩ)															
	eA-gA	eA-gB	eA-gC	eA-gD	eA-gE	eB-gA	eB-gB	eB-gC	eB-gD	eB-gE	eC-gA	eC-gB	eC-gC	eC-gD	eC-gE	ECG
100 Hz–10 kHz	20.45	19.42	8.24	24.35	23.80	19.81	18.84	10.92	25.95	23.81	23.07	19.70	20.52	21.25	25.90	19.93
10 kHz–1 MHz	1.21	0.39	0.64	1.61	0.52	0.77	0.62	0.48	0.80	0.86	0.70	0.95	0.42	0.40	0.41	0.73
100 Hz–1 MHz	21.66	19.81	8.88	25.96	24.32	20.58	19.45	11.39	26.76	24.67	23.77	20.65	20.93	21.65	26.31	20.66

*ECG* electrocardiogram

**Fig. 4:**
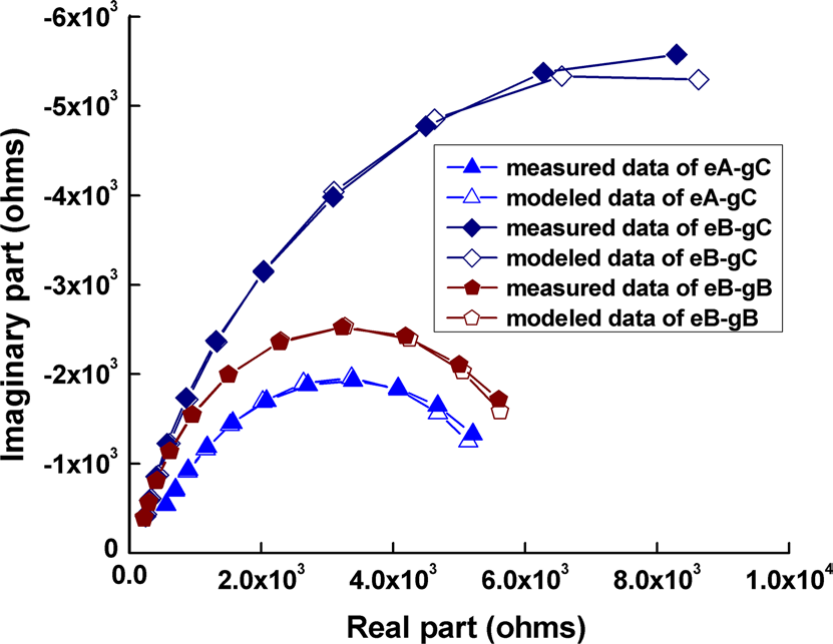
Measured and modeled values of the eA-gC, eB-gC, and eB-gB electrode–conductive gel combinations

A simplified Cole system electrical equivalent model was employed to parameterize our results, which was represented by a resistor *R*_*s*_ (associated with the resistance of the conductive gel and sweat) in series with a parallel combination of a constant phase element (CPE) and a resistor *R*_*d*_ (determined by the property of the stratum corneum between the metal electrode and the underlying tissue) [24, 31]. The mean contact impedance values of the combination eA-gC (WHU CX-150-02 and Greentek GT5, which are Ag^+^/Ag^+^Cl^−^ powder electrode made by mixing silver particles and silver chloride particles and conductive gel with low viscosity), eB-gC (Greentek BX-RL02-1500 and Greentek GT5, which are Ag^+^/Ag^+^Cl^−^ powder electrode created by plating a uniform silver chloride on a silver substrate and conductive gel with low viscosity), and eB-gB (Greentek BX-RL02-1500 and Greentek GT10, which are Ag^+^/Ag^+^Cl^−^ powder electrode created by plating a uniform silver chloride on a silver substrate and a water-based polymer conductive gel) were modeled (Fig. 4) because these three combinations had relatively low contact impedance values (see Table 1). Moreover, because the impedance of the underlying tissues (~ 100 Ω) was negligible compared with the contact impedance (generally > 1 kΩ) across the 100 Hz–10 kHz frequency range [20, 32], only the contact impedance across the 100 Hz–10 kHz was modeled. The Chi square goodness of fit test for these three combinations was < 0.0001, indicating a good fitness (see Table 2). Table 2 shows that the *R*_*d*_ value for the eB-gB combination (14,670 Ω) was much larger than that for the eA-gC (6138 Ω) and eB-gB (6399 Ω) combinations, indicating a high resistance from the stratum corneum. This may be why the contact impedance value of the eB-gB combination was larger than that of the eA-gC and eB-gB combinations. In addition, the *Y*_0_ value for the eB-gB and eB-gC combinations was similar, but clearly different from that for the eA-gC combination.

**Table 2:** The three circuit components R_s_, Y_cpe,_ and R_d_ for the eA-gC, eB-gC, and eB-gB electrode–conductive gel combinations

Combination	Rs (ohm)	CPE		Rd (ohm)	Chi square
		Y_0_ (S s^*n*^)	*n*		
eA-gC	241.5	6.02E−07	0.7106	6138	2.82E−04
eB-gB	106	3.08E−07	0.8051	14,670	8.63E−04
eB-gC	104.1	2.08E−07	0.8486	6399	4.46E−04

*CPE* constant phase element

## Discussion

EIT has shown promise as a sensitive medical imaging tool to monitor brain injury long-term and detect stroke rapidly, thus making the timely intervention possible and improving the prognosis of patients. However, brain EIT is unavoidably influenced by electrode–skin contact impedance because the contact impedance significantly varies with time and frequency. Thus, it is highly desirable for brain EIT to use the combination of electrode and conductive gel that performs best with both time and frequency. Accordingly, in this study, the contact impedance of 16 combinations of electrode and conductive gel, consisting of four kinds of clinical electrode and five types of commercially available conductive gel, was measured on ten healthy volunteers’ forehead/scalp using two-electrode strategy for a period of 1 h at frequencies from 100 Hz to 1 MHz. And then according to the measurement results, the overall performance of each combination was systematically evaluated regarding the magnitude of contact impedance, and the change in contact impedance with time and frequency.

### Magnitude of contact impedance

As shown in Fig. 1b, the magnitude of contact impedance of all combinations dramatically decreased from tens of kohms to hundreds of ohms over the whole frequency range, especially within 100 Hz–100 kHz, which largely agrees with the results published in other studies [20, 25]. Additionally, Goren et al. recently collected the quality multi-frequency EIT (MFEIT) data from real stroke patients and pointed out that the electrode contact was acceptable if the electrode contact impedance was less than 3 kΩ at 1 kHz [33]. For validation, the measured results of all combinations at 1 kHz were compared with 3 kΩ. It is found that the mean contact impedance of most combinations is less than 3 kΩ, except for the combination eA-gA (about 4000 Ω) and eA-gD (about 5100 Ω), indicating that our measured results are reliable.

In this study, the two-electrode method was employed to measure impedance because the measured results contained the electrode–skin contact impedance. In normal EIT practice, the four-electrode technique was always applied in EIT data collection in order to alleviate the effects of contact impedance by assuming that the EIT system has ideal performances, such as a high (ideally infinite) input impedance and an unlimited common-mode rejection ratio (CMRR). But in practice, neither of these ideal elements can be perfectly realized; thus the non-ideal performances make the accuracy of EIT data measurement adversely affected by contact impedance. First, high contact impedance can give rise to a certain potential difference at the electrode–skin interface, which may hide the impedance changes of the underlying tissues [34]. For instance, Fig. 1b shows contact impedance could reach up to 30 kΩ at 100 Hz, which is far larger than the impedance of the underlying tissues (several hundred ohms), but was comparable with the input impedance of the EIT system (dozens of Mohms [20]). Second, the imbalance contact impedance between two electrodes can also lead to measurement errors by converting the common mode voltage to a differential mode voltage at the amplifier input of EIT system [35]. Yang et al. found that the imbalance between two measuring electrodes can cause more than 0.1% error in measurement and significant artifacts in EIT images [20]. In fact, it is difficult to ensure a balanced contact impedance between two electrodes when applying the electrodes. Therefore, it is essential to evaluate the magnitude of electrode–skin contact impedance so as to determine the optimal combination of electrode and conductive gel with the lowest possible contact impedance. The results of this study showed that the eA-gC combination clearly had a low contact impedance within 100 Hz–10 kHz and the contact impedance of the eB-gC combination was relatively small within 10 kHz–1 MHz (Fig. 1b), indicating that the eA-gC and eB-gC combinations may be the most appropriate for EIT measurement.

Additionally, it was found that the combinations that used conductive gel C (Ten 20^®^), such as the eA-gC, eB-gC and eC-gC combinations, had relatively low impedance compared with the combinations using other types of conductive gel. This may due to the mechanical properties of conductive gel. Conductive gel C is a low viscosity gel while other types of gels have higher viscosity. Thus, gel C is more likely to fill in any non-uniform areas on the forehead/scalp, resulting in an increased surface (contact) area. Naturally, an increased contact area leads to a decreased in impedance [36].

Furthermore, several factors affect the accuracy of EIT data, such as skin abrasion, pressure between electrode and scalp, patient motion, temperature, site and the condition of skin [37]. In this study, to avoid the effects of these factors, the following measures were taken. First, before measurement, the forehead/scalp was cleaned with an alcohol solution to remove the skin grease and the medical tape was employed to maintain a good connection between electrode and forehead/scalp. Second, for each subject, a bandage with 1.5 times the head circumference of each volunteer was wrapped around the head twice to keep the pressure between electrode and scalp stable. Third, all volunteers were asked to keep still throughout the experiments to avoid motion artifacts. Fourth, all measurements were carried out under a relatively stable ambient temperature (25 ± 1.5 °C). Fifth, all combinations were applied to measure impedance at the same site on each volunteer. Finally, the interval between measurements of two adjacent combinations was set to 2 days in order to restore the condition of forehead/scalp to the previous state and thus ensure that conditions of forehead/scalp are similar for measurements of all combination.

### Change in contact impedance with time

Because the current of 50 kHz is usually used in brain EIT for long-term monitoring of brain injury, the magnitudes of contact impedances of all combinations at 50 kHz were recorded and compared as soon as the electrodes were applied. It was found that the eB-gC combination had a relatively low impedance (about 300 Ω, Fig. 2a) but there was no significant difference in impedance between eB-gC and the other combinations, except for eA-gD, indicating that there was no obvious discrepancy in contact impedances of all combinations (except eA-gD) when the electrodes were immediately attached. This may because the same procedure of electrode application was carried out before measuring the impedance of each combination in this study. Using the same procedure ensured the same conditions were maintained at the electrode–paste–scalp interface for each combination; such conditions included the thickness of the stratum corneum and the pressure between the electrode and forehead/scalp, which are major contributors to electrode–skin contact impedance.

Because brain EIT may be used for long-term monitoring, it is essential to select the optimal combination of electrode and conductive gel, that is, one that provides stable contact impedance with time. The results of this study showed that the contact impedance of each combination varied over time, indicating that contact impedance is a function of time. The eA-gC combination showed a significantly small change in contact impedance for 1 h, suggesting that it may be the best combination for long-term monitoring with brain EIT. Additionally, it is found that the change in contact impedance decreased over time. A plausible explanation would be that the conditions between electrode and forehead/scalp tended to be stable over time. For example, the temperature of the conductive gel gradually increased and normalized with body temperature and the sweat ducts of the forehead/scalp gradually stabilized [32]. Furthermore, the change in contact impedance during the first 10 min after attaching the electrodes was about 2–3 times greater than that recorded during other time periods, suggesting that the impedance should be measured for 10 min after the electrodes have been attached to reduce the effects of a change in contact impedance with time when EIT is used in long-term monitoring. Additionally, in brain EIT data collection, the electrode–skin contact impedance directly affects the accuracy of measurement because electrodes attached to human body are used for both current injection and boundary voltage measurement. Especially in long-term monitoring, the contact impedance will inevitably change over time due to variations in the conditions (such as temperature and humidity) of electrode–skin interface, resulting in the changes of contact impedance that are avoidably contained by the collected EIT data. Thus, in order to evaluate the data quality, real-time monitoring of contact impedance is crucial in brain EIT data collection.

### Change in contact impedance with frequency

Change in contact impedance with frequency is the major concern when using brain EIT to detect acute stroke because brain impedance spectroscopy is needed. In an ideal scenario, electrode–skin contact impedance remains constant across frequency; the measured impedance spectra are only attributed to the underlying tissues. Therefore, a combination of electrode and conductive gel with a minimum change in contact impedance with frequency is preferable. The results of this study demonstrated that the eA-gC combination performed best regarding a change in contact impedance with frequency (see Table 1).

In this study, the electrode–conductive gel–scalp interface was simplified by using a Cole model consisting of three components. This was used to model the impedance spectra of the three combinations with the smallest contact impedance change: eA-gC; eB-gB; and eB-gC. The *R*_*d*_ values of the eA-gC and eB-gC combinations were close but far smaller than that of the eB-gB combination. This may be due to the difference in conductive gel used in the three combinations. Conductive gel C had low viscosity and it may have penetrated the stratum corneum, leading to a decrease in resistance of the stratum corneum, namely *R*_*d*_. In addition, the *Y*_0_ parameters of the eB-gB combination were close to those of the eB-gC combination, but they were clearly different from those of the eA-gC combination. This may be because polarizable electrodes accumulate charges at the electrode–scalp contact site.

### Limitations and technical considerations

For the consideration of the clinical relevance of our data, the data measurement time should be taken into account. In this study, the time property of the contact impedance of all 16 combinations was evaluated within 1 h after the electrodes had been attached to the forehead/scalp of each volunteer. This period of time was far shorter than that used in actual EIT monitoring (several hours or even days) [11, 33]. However, our research is still meaningful in terms of significant implications regarding the selection of combination of electrode and conductive gel. First, the change in contact impedance during the first 10 min after attaching the electrodes was about 2–3 times greater than that recorded afterwards, suggesting that the impedance should be measured 10 min after the electrodes are attached so that the effects of a change in contact impedance with time will be alleviated in long-term monitoring using EIT. Second, the combination of Ag^+^/Ag^+^Cl^−^ powder electrode made by mixing silver particles and silver chloride particles and conductive gel with low viscosity (eA-gC) had a significantly smaller change in contact impedance within 1 h (*p *< 0.05), which may provide an effective benchmark when selecting the appropriate combination of electrode and conductive gel to be used in the long-term monitoring of brain injury using EIT. Furthermore, skin preparation before attaching electrodes should also be considered because it is an essential procedure in clinical EIT application. In this study, the forehead/scalp was cleaned to remove the skin grease: after 75% alcohol solution was applied to each electrode site with a medical cotton swab, each electrode site was repeatedly rubbed using medical gauze. We did not use the special skin preparation gel with the function of abrasion such as Nuprep gel (Weaver and Co., USA) used by Goren et al. [33]. However, during the skin cleaning in our study, the skin grease was effectively removed and some epidermis was also stripped away, which was equivalent to the outcome of skin abrasion to a certain extent. Most importantly, the contact impedance of most combinations was below 3 kΩ and compared to the results of Goren et al. [33], which indicated that our electrode application process (including skin preparation with alcohol solution) was valid. Moreover, by using the same electrode application process as in this study, our group have carried out some clinical research with encouraging results, such as the use of EIT to monitor regional cerebral edema during clinical dehydration treatment [11] and cerebral impedance change during total aortic arch replacement [28], suggesting the clinical relevance of the measurement results in this study.

Moreover, the results presented in this study were obtained from healthy and young adults (age range: 19–28 years), whereas brain EIT is more often used to monitor and detect stroke in older patients. It has been shown that the number of stratum corneum layers increases with age, leading to higher stratum corneum resistance (*R*_*d*_ in this study) [38]. But it is reasonable to apply the findings of this study to older patients because stratum corneum resistance is the same for all combinations of electrode and conductive gel.

In this study, the two-electrode strategy was used to measure contact impedance. Through this method the brain impedance cannot be separated from the electrode–skin contact impedance, making the measured results include the brain tissue impedance. However, measurements of all combinations were carried out on identical sites of each volunteer’s forehead, ensuring the same tissue impedance for all combinations. Therefore, it is theoretically appropriate to evaluate the performance of all combinations using the measurement results of the two-electrode method. Additionally, the reasons why the two-electrode technique is employed are as follows. First, this method can be implemented most easily compared with other methods such as the three-electrode approach [39, 40] and the improved four-electrode method [41], and it has been widely used in the measurement of electrode–skin contact impedance [21, 33]. Second, in order to eliminate the effect of tissue impedance when comparing contact impedance performance of all combinations of electrode and conductive gel, the measurements should be performed on identical sites for each volunteer. Because two measurement sites are needed in only two-electrode method, applying this method can best ensure that the tissue impedance is the same for all measurements.

In this study, in order to improve the EIT data quality and reduce the effect of contact impedance, we focused on the impedance property of electrodes and conductive gel, which is the most important factor affecting contact impedance because EIT data is collected through electrode and conductive gel. We also attempted to select an optimal combination of electrode and conductive gel that had superior performance on contact impedance change over time and with frequency. Additionally, some novel EIT image reconstruction algorithms were proposed to alleviate the impact of contact impedance, in which the complete electrode model (CEM) was used to simultaneously recover the contact impedance and interior impedance of the body. For example, Vilhunen et al. formulated the inverse problem as one of Bayesian estimations based on finite element approximation [42, 43]; Demidenko et al. relied on the analytical solution using a Neumann-to-Dirichlet matrix to estimate the contact impedance separately [44, 45]; Boverma et al. employed linear–algebraic manipulations to simultaneously recover time-varying contact impedance and the interior impedance [46, 47]. Experimental results showed that the EIT image artifacts due to contact impedance were successfully reduced. Therefore, in the future, approaches to select electrode and conductive and image reconstruction algorithms to reduce the influence of contact impedance are both recommended to be used in actual EIT applications.

Low frequency currents (< 1 kHz) were used in this study, which might cause skin perception or pain. International Electrotechnical Commission (IEC) specifies a “patient auxiliary current” limit of 100 μA from 0.1 Hz to 1 kHz, but IEC also points out that the application of larger currents is permitted for diagnostic purposes [48]. According to the research results of Romsauerove et al. [49], the constant voltage exciting mode (500 mV) was used to ensure safety in this study. Normally, the largest current would be obtained with the smallest contact impedance. Thus, by choosing the smallest contact impedance at upper and lower limit of measurement frequency (5.7 kΩ at 100 Hz and 2.1 kΩ at 1 kHz, respectively), we calculated the current to be 87 μA and 238 μA, which were still within the current limits (280μA above 100 Hz) suggested by Romsauerova et al. for stroke diagnosis using EIT. Most importantly, in all experiments of our study, we assured the volunteers ahead that we would immediately terminate the experiment as soon as they felt uncomfortable. As expected, none of the subjects reported discomfort. Therefore, the constant voltage exciting mode (500 mV) we applied was safe.

## Conclusions

In this study, the contact impedance of 16 electrode–conductive gel combinations, consisting of four types of clinical electrodes and five types of commercially available conductive gel, was measured using the two-electrode method, and then the overall performance of all combinations was compared in terms of the magnitude of contact impedance, and changes in contact impedance with time and frequency. Results showed that the eA-gC combination, which is the combination of the Ag^+^/Ag^+^Cl^−^ powder electrode and low viscosity conductive gel (Ten 20^®^), had a relatively low magnitude of contact impedance and offered superior performance regarding contact impedance with time (*p* < 0.05) and frequency (*p* < 0.05), suggesting that the combination of Ag^+^/Ag^+^Cl^−^ powder electrode and low viscosity conductive gel may be the optimal candidate for use in brain EIT. Future studies should focus on the practical use of the eA-gC combination when EIT is used to monitor brain injury long-term and/or rapidly detect stroke. It is hoped that this study provides a basis for selecting the optimal electrode–conductive gel combination for use in brain EIT.

## Method

### Electrodes

**Fig. 5:**
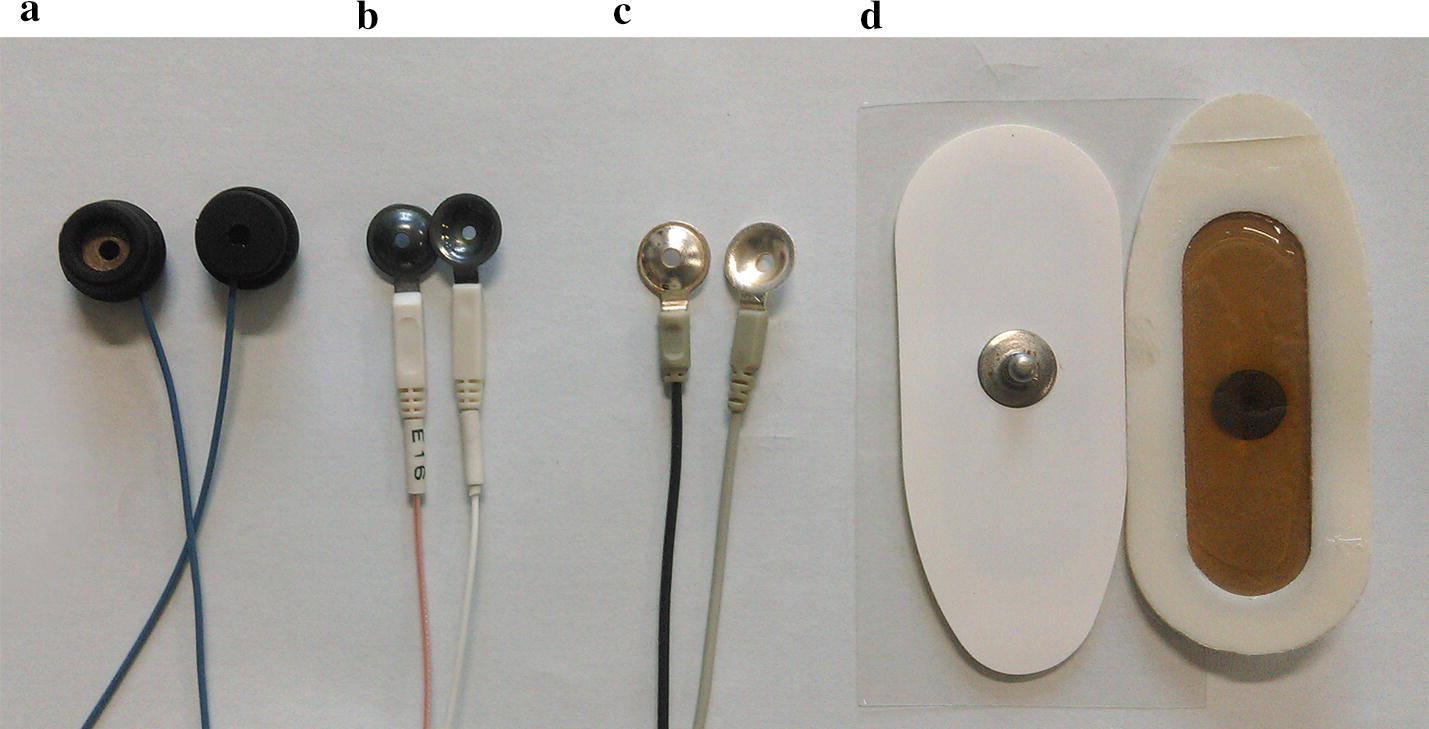
The four types of electrodes investigated in the study

Four kinds of electrodes, which are typically used within clinical setting, were investigated in this study (Fig. 5): Ag^+^/Ag^+^Cl^−^ powder electrode; Ag^+^/Ag^+^Cl^−^ plating electrode; silver electrode, and electrocardiogram (ECG) electrode. Bearing in mind the characteristics of the nearly perfect non-polarization of the Ag^+^/Ag^+^Cl^−^ electrode and its low electrode–skin contact impedance, two types of Ag^+^/Ag^+^Cl^−^ electrode were selected; these are the most commonly used and preferred to record biological signals in clinical measurements. Moreover, because of good conductivity, high precision, low electrical noise and stable polarization potential of the silver electrode, a silver electrode (with a purity of 99.99%) was also chosen; this is widely used in electroencephalogram (EEG) signal acquisition. Because the ECG electrode is often employed in EIT, a disposable ECG electrode was also studied.

Electrode A is an Ag^+^/Ag^+^Cl^−^ powder electrode (CX-150-02, College of Chemistry and Molecular Sciences, Wuhan University, Wuhan, China); it is made by mixing silver particles and silver chloride particles. Electrode B is also an Ag^+^/Ag^+^Cl^−^ powder electrode (BX-RL02-1500, Wuhan Greentek Technology Pty. Ltd, Wuhan, China), but it is created by plating a uniform silver chloride on a silver substrate. Electrode C is a silver electrode (GT2015012, Wuhan Greentek Technology Pty. Ltd, Wuhan, China). Electrode D is a self-adhesive ECG electrode with the conductive gel (Shanghai Shenfeng Medical & Health Articles Co., Ltd, Shanghai, China). All electrodes had a 10-mm diameter.

### Conductive gel

**Fig. 6:**
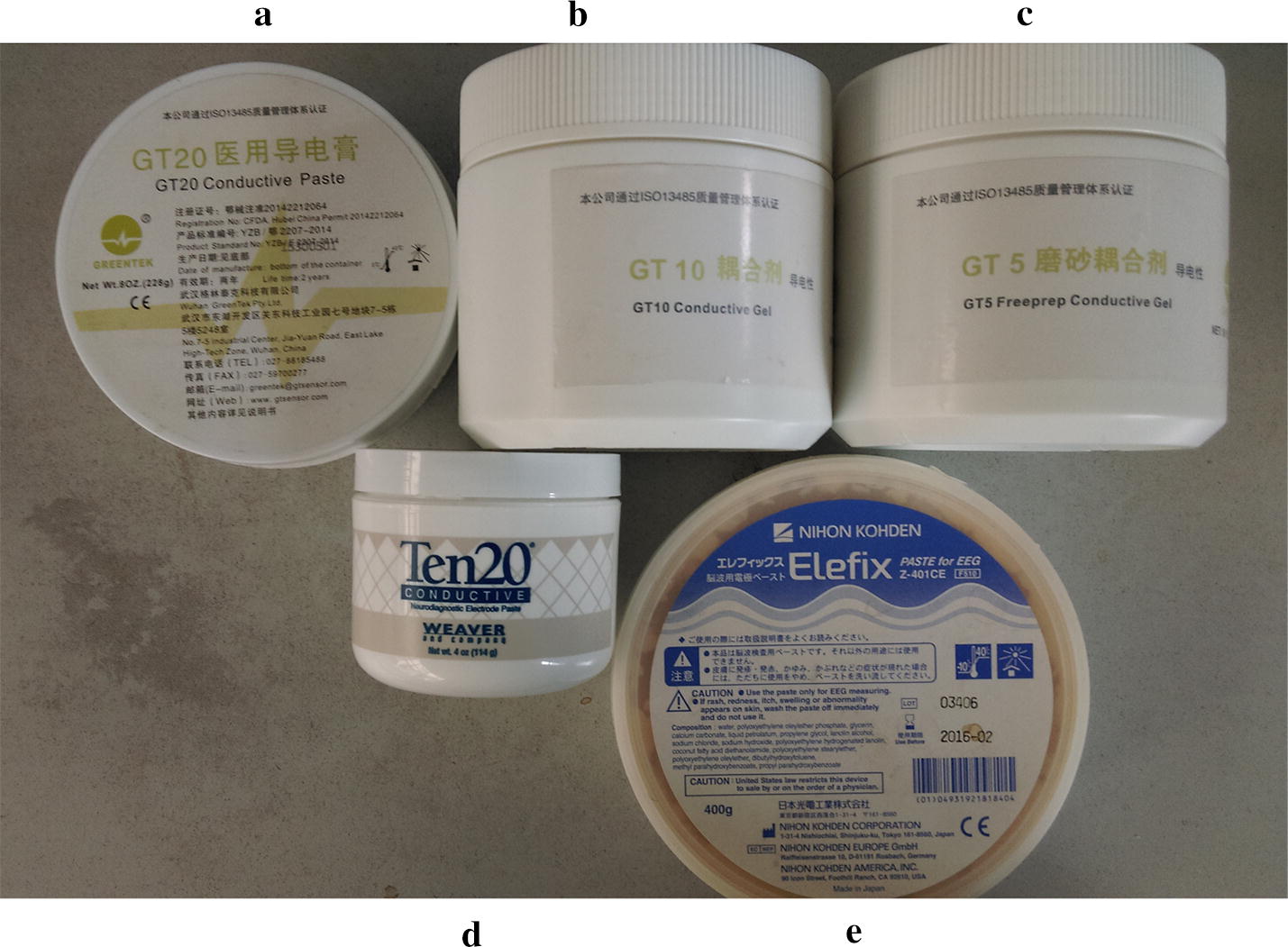
The five types of conductive gels investigated in the study

Five types of conductive gels commonly used in EEG signal acquisition were studied (Fig. 6). Conductive gel A is a medical EEG conductive gel with good conductivity and moisture retention; it can maintain low impedance for a sustained period (GT20, Wuhan Greentek Technology Pty. Ltd, Wuhan, China). Conductive gel B is a water-based polymer conductive gel compounding with waster-based polymer and skin-friendly medical materials; it has high conductivity and quickly reduces any electrode–skin contact impedance (GT10, Wuhan Greentek Technology Pty. Ltd, Wuhan, China). Conductive gel C is a medical conductive gel; it not only has the function of conductivity but also can reduce the electrode–skin contact impedance by removing the stratum corneum and skin grease (GT5, Wuhan Greentek Technology Pty. Ltd, Wuhan, China). Conductive gel D has good viscosity and conductivity; this ensures that the biological signal is transmitted stably and accurately (Ten20, Weaver and company, Denver, Colorado, USA). Conductive gel E is a commercial EEG electrode gel (Elefix, Z-410CE; Nihon Kohden, Tokyo, Japan).

Several clinical applications of these types of conductive gel have demonstrated that they have good conductivity; however, there is significant difference in viscosity between them. Gel A, D and E have similar viscosity, being made of solid paste. Gel C has the lowest viscosity, being a low viscosity paste. Gel B has a viscosity between paste A and C.

### Study cohort

All experiments conducted in this study were approved by the Ethics Committee of the Fourth Military Medical University, Xi’an, People’s Republic of China; all volunteers signed a written informed consent form.

Each electrode type was combined with each type of conductive gel for a total of 16 combinations, except for the disposable ECG electrode which is self-adhesive and has its own conductive gel. All combinations were applied to the forehead/scalp of ten healthy adult volunteers to measure electrical impedance (five women and five men; age 20 ± 5.3 years; weight 60 ± 10.8 kg). All experiments were carried out at an ambient temperature of 25 ± 1.5 °C; humidity was kept at 50 ± 5%. Volunteers were asked to lie down in a supine position and hold as still as possible.

### Electrical impedance measurement

#### Hardware and protocol

**Fig. 7:**
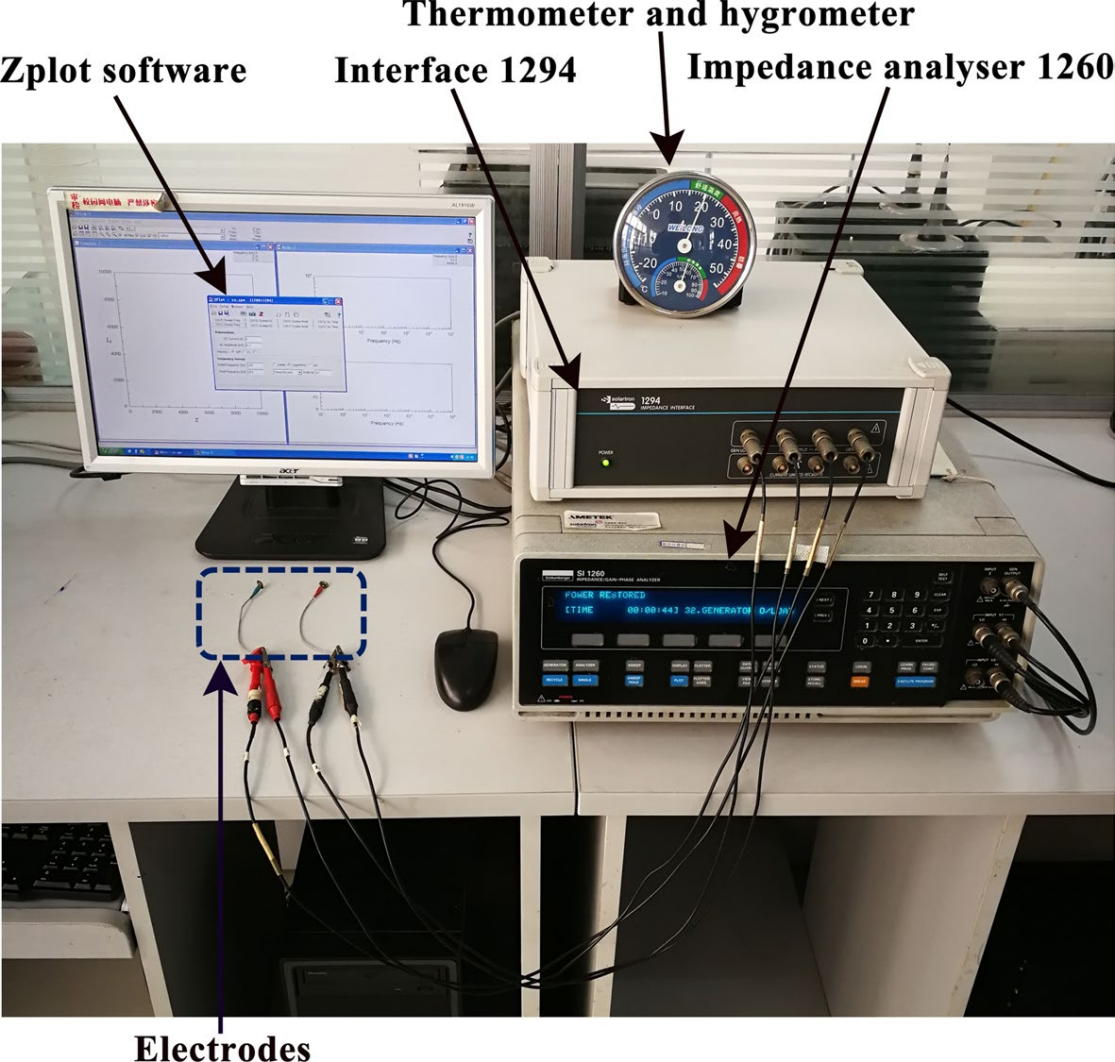
Electrical impedance measurement system

The electrical impedance measurement set-up employed in this study consisted of three parts: the Solartron1260 impedance/gain-phase analyzer (Solartron Analytical, Farnborough, UK), the 1294A impedance measurement interface system (Solartron Analytical, Farnborough, UK), and the ZPlot^®^ software (Scribner Associates Inc., Southern Pines, NC, USA). The software was used to control the acquisition parameters and was applied on a desktop computer (2.3 GHz processor, Windows XP), as shown in Fig. 7.

To evaluate the time property of contact impedance, the impedance measurement was performed at 10-min intervals for 1 h after electrodes were applied. To assess the frequency property of contact impedance, the impedance spectra measurement within the frequency range from 100 Hz to 1 MHz (5 points per decade) was conducted. For safety reasons, the constant voltage exciting mode (500 mV) was used and volunteers were assured beforehand that the experiment would be terminated immediately if they felt uncomfortable.

**Fig. 8:**
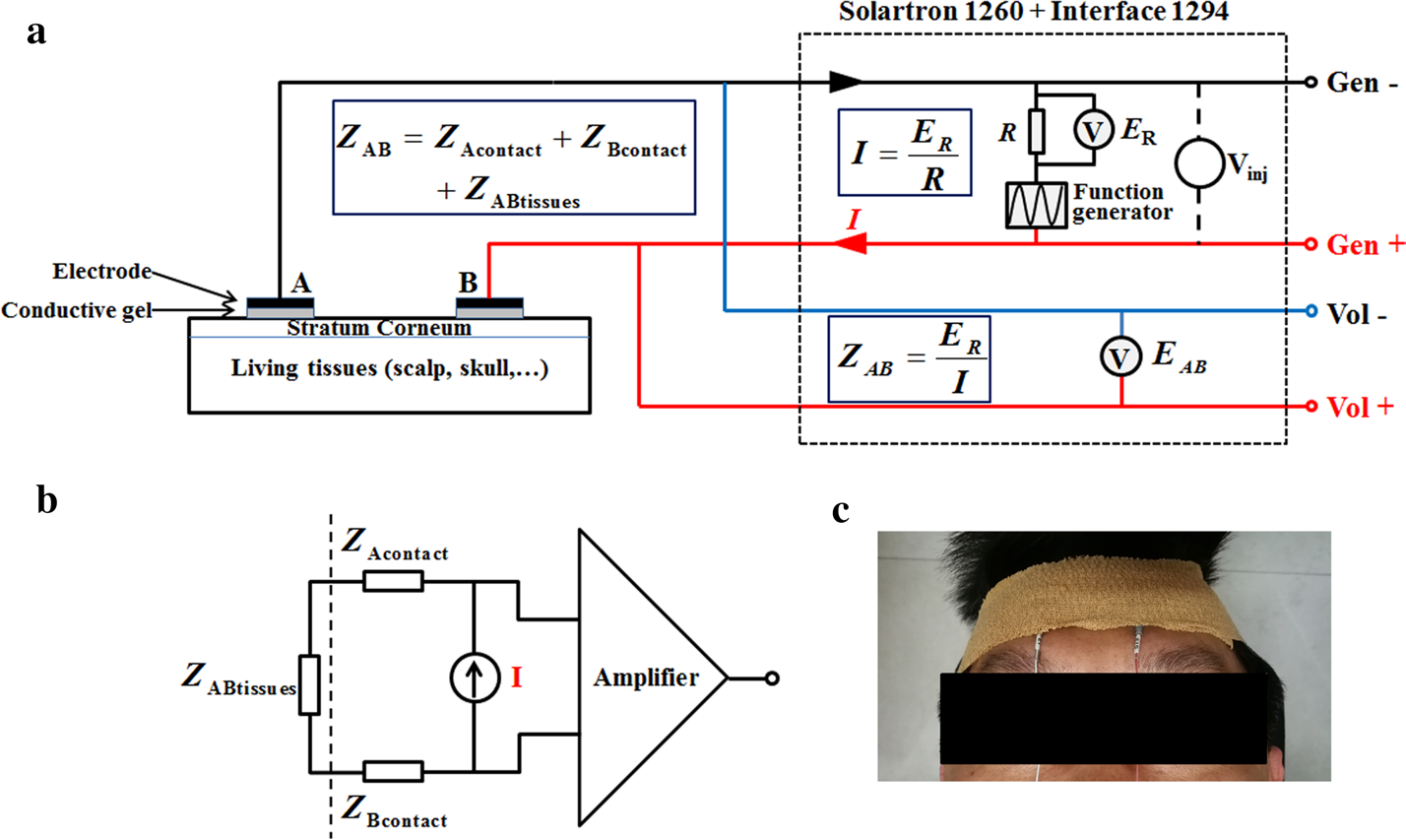
**a** Experimental set-up. **b** Simplified schematic diagram of the two-electrode technique. **c** Placement of electrode on the forehead

Theoretically, to compare the performance of all combinations of electrode–conductive gel, the accurate electrode–skin contact impedance should be measured. However, in practice, contact impedance and the underlying tissue impedance always mixed together; so far, there is no effective way to separate them [19, 20, 32]. Therefore, in this study, the two-electrode strategy was applied to perform impedance measurement because the measured results (*Z*_AB_) of the two-electrode method contain not only the tissue impedance (*Z*_AB-tissue_) between the two electrodes, but also the electrode–skin contact impedance (*Z*_A-contact_ and *Z*_B-contact_), i.e. *Z*_AB_ = *Z*_A-contact_ + *Z*_AB-tissue_ + *Z*_B-contact_, as shown in Fig. 8a and b [39]. In the two-electrode method, the two electrodes serve both as exciting electrodes and as measuring electrodes. The electrode positions are shown in Fig. 8c and electrodes were spaced 3 cm apart. To eliminate the effect of tissue impedance between two electrodes when comparing the contact impedances of all combinations, all measurements were carried out on a identical site of the forehead for each volunteer, thus ensuring the same tissue impedance for all combinations.

The experimental procedure was as follows (see also Fig. 7):

1. The forehead was cleaned with 75% alcohol solution before an electrode–conductive gel combination was fixed to the scalp with medical tape (3 M Corporation, St. Paul, MN, USA).
2. A medical bandage (McDavid-4575, Bellwood, USA) with 1.5 times the head circumference of each volunteer was wrapped around the head twice to further fasten the electrode; impedance spectra were measured immediately, and then after 10, 20, 30, 40, 50 and 60 min, respectively. A total of seven measurements were obtained.
3. The electrode–conductive gel combination was removed from the scalp; the same procedure (steps 1 and 2) was used with the remaining nine subjects.
4. After 2 days, steps 1–3 were repeated using a different electrode–conductive gel combination.

In brief, for each volunteer, seven impedance spectra measurements as a function of time and frequency for each kind of electrode–conductive gel combination were obtained. Additionally, to eliminate random errors, measurements were obtained at every time point three times; the average of these three measurements was taken as the final result.

#### Resistor measurement

To test system performance, the impedance of three high-precision resistors (250 Ω, 2 kΩ, 15 kΩ) was recorded and then compared with the results obtained using a digital multimeter (Fluke 15B, Fluke Corporation, Everett, WA, USA). Before each experiment, the impedance of each resistor was measured and used as a control.

#### Performance criteria and data analysis

In general, the magnitude of contact impedance, the stability of the signal, the signal to noise ratio, and the uniformity of signals of the different electrodes were utilized to directly evaluate the electrode performance and indirectly reflect the contact impedance. However, when brain EIT is used to monitor brain injury long-term and/or rapidly detect stroke, these performance criteria are not suitable to choose an optimal combination of electrode–conductive gel because the time and frequency properties of contact impedance cannot be evaluated using these criteria. Instead, in this study, the magnitude of contact impedance, and changes in contact impedance with time and frequency were proposed to comprehensively assess the time and frequency properties of contact impedance.

#### Magnitude of contact impedance

In EIT data acquisition, the measured impedance generally consists of two parts (as shown in Fig. 8a): the contact impedance and the underlying tissue impedance. Especially at the low frequency range (< 1 kHz), contact impedance can reach up to tens of kohms and thus dominate the measured results. Although contact impedance decreases with frequency, it can still be hundreds of ohms at high frequencies (> 100 kHz) [20, 32], which is comparable to tissue impedance (about 100 Ω). And thus, contact impedance inevitably affects the accuracy of measured impedance in practice. Therefore, it is desirable for the combination of electrode and conductive gel to have the smallest possible electrode–skin contact impedance. In this study, the difference in measured impedance within the low (100 Hz–10 kHz) and high (10 kHz–1 MHz) frequency ranges was compared.

Since the module of impedance (|*Z*|) is used in most EIT applications, |*Z*| was calculated according to the results (real (*Z*_real_) and imaginary (*Z*_img_) part of impedance) obtained with the Solartron 1260 impedance/gain-phase analyzer.

#### Change in contact impedance with time

Long-term monitoring of brain injury with EIT is often carried out for several hours or days. As a result, the extent of the change in contact impedance with time directly affects the precision of the data obtained. In this study, first, the magnitude of impedance of all combinations at 50 kHz and 1 kHz was respectively compared because currents of 50 kHz and 1 kHz are used in most EIT applications involving long-term monitoring brain injury [17, 27–29].

Second, the measured values of all the combinations were normalized by the largest value of mean impedance to reduce any variability between individuals. In addition, the entire time range was divided into six sub-time frames (each sub-time frame lasting 10 min); the change in impedance of each combination during each sub-time frame was calculated thus:

1 $$ \Delta Z_{i} = Z_{10(i + 1)} - Z_{10i} \quad (i = 1,2, \ldots ,6) $$

where *i* denotes the *i*st time range, $\Delta Z_{i}$ represents the impedance change during the $i{\text{st}}$ time range, and $Z_{10i}$ and $Z_{10(i + 1)}$ represent the impedance at time points $10i$ and $10(i + 1)$, respectively.

Finally, a score was calculated by adding the change in impedance for each combination during all sub-time frames together. The smaller the score, the better the time property of the electrode–conductive gel combination.

The IBM SPSS Statistics for Windows software (version 22, IBM Corporation, Armonk, NY, USA) was used to analyze the data; data were compared with a one-way analysis of variance (ANOVA). A post hoc test was used and *p* < 0.05 was deemed statistically significant.

#### Change in contact impedance with frequency

Based on the fact that the impedance (frequency) spectra of stroke lesions differ from that of normal brain tissues, EIT used this difference to detect stroke [16, 50]. But it is noted that contact impedance changes significantly with frequency [32]; thus, it also adversely affects the accuracy of measured impedance spectra. In order to reduce the effect of contact impedance, a combination of electrode and conductive gel with a relatively small change in contact impedance with frequency is preferred.

In this study, on the one hand, the changes in impedance of all the electrode–conductive gel combinations across the 100 Hz–1 MHz frequency range were calculated as follows:

2 $$ \Delta Z = Z_{ \text{max} } - Z_{ \text{min} } $$

where *Z*_max_ and *Z*_min_ are the maximum and minimum mean impedance values for the 100 Hz–1 MHz range, respectively. The changes in impedance of all the electrode–conductive gel combinations were compared with a one-way ANOVA.

**Fig. 9:**
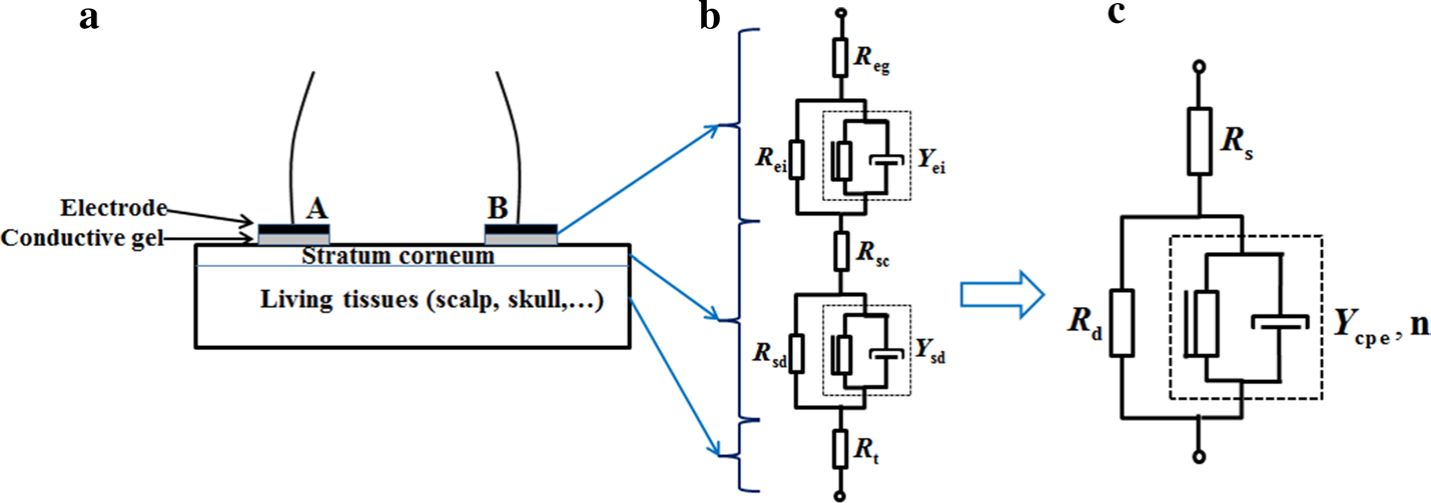
Composite of the electrical equivalent model of electrode–skin-underlying tissue. **a** Electrode–skin-underlying tissue composite. **b** Equivalent model of electrode–skin-underlying tissue. The electrode–conductive gel interface is represented by the resistor $R_{\text{eg}}$ in series with a parallel combination of a constant phase element (CPE) $Y_{\text{ei}}$ and a resistor $R_{\text{ei}}$. The stratum corneum is represented by a resistor $R_{\text{sc}}$ in series with a parallel combination of a CPE $Y_{\text{sd}}$ and a resistor $R_{\text{sd}}$. The underlying (living) tissue is modeled by a resistor $R_{\text{t}}$. **c** The simplified Cole system electrical equivalent model of electrode–skin interface because the impedance of the electrode–conductive gel interface is much smaller than that of the stratum corneum [20]. This model is represented by a resistor $R_{s}$ in series with a parallel combination of a CPE and a resistor $R_{d}$. $R_{s}$ is associated with the resistance of the wire, conductive gel, and sweat. CPE and $R_{d}$ are determined by the property of the stratum corneum between the metal electrode and the underlying tissue. CPE is the capacitance of the stratum corneum denoted by $Y_{cpe} = Y_{0} (j\omega )^{n}$, where $\omega$ is the angular velocity, $Y_{0}$ is the magnitude of CPE at $\omega = 1$, *n* is a constant, *j* is the imaginary unit $\sqrt { - 1}$, and $R_{d}$ is the resistance of the stratum corneum

On the other hand, to further analyze the difference in impedance of the different electrode–conductive gel combinations, the impedance spectra were parameterized using a simplified Cole system electrical equivalent model [51–53], as shown in Fig. 9. In this study, the three combinations with a relatively small change in impedance across the whole frequency range were selected; their impedance spectra were divided by two to obtain the impedance spectrum of one single electrode under the assumption that the impedance properties of two electrodes were same. This assumption is reasonable because, in this study, the two electrodes were made of the same material and were of same size. Then the impedance spectrum of the single electrode was modeled using the ZSimpWin software (version 3.1, Princeton Applied Research, Oak Ridge, TN, USA).

## Abbreviations

EIT: electrical impedance tomography
CT: computed tomography
MRI: magnetic resonance imaging
CMRR: common-mode rejection ratio
CPE: constant phase element
IEC: international electrotechnical commission
